# Challenges and Management of Acute Coronary Syndrome in Cancer Patients

**DOI:** 10.3389/fcvm.2021.590016

**Published:** 2021-06-09

**Authors:** Isabela Bispo Santos da Silva Costa, Fernanda Thereza de Almeida Andrade, Diego Carter, Vinicius B. Seleme, Maycon Santos Costa, Carlos M. Campos, Ludhmila Abrahão Hajjar

**Affiliations:** ^1^Cancer Institute University of São Paulo, São Paulo, Brazil; ^2^Heart Institute (InCor), University of São Paulo Medical School, São Paulo, Brazil; ^3^Hospital Santana - Mogi das Cruzes, Mogi das Cruzes, Brazil

**Keywords:** acute coronary syndrome, cancer, cardiotoxicity, coronary disease, cardio oncology

## Abstract

Cancer and cardiovascular disease are the leading causes of mortality in the world. The prevalence of cardiovascular risk factors and coronary artery disease in cancer patients is elevated, and it is associated with high mortality. Several mechanisms, such as the proinflammatory and procoagulant states present in cancer patients, may contribute to these scenarios. Oncological therapy can predispose patients to acute thrombosis, accelerated atherosclerosis and coronary spasm. Treatment decisions must be individualized and based on the cancer history and balancing bleeding and thrombosis risks.

## Background

Cancer and its treatment are highly associated with coronary artery disease (CAD), which is an important cause of mortality in cancer survivors (1–4). The state of chronic inflammation present in cancer patients, which is associated with their predisposition to present arterial and venous thromboembolic events, may favour adverse events in the cardiovascular system (5, 6). In addition, several oncological therapies may cause severe coronary lesions due to the predisposition to accelerated atherosclerosis, endothelial dysfunction, acute coronary thrombosis and coronary spasm (7).

Notably, cancer and CAD share several risk factors, such as hypertension, diabetes, obesity, sedentarism, and smoking. Moreover, some factors commonly present in cancer patients, such as anaemia, hypoxaemia and hyperviscosity, are factors that trigger acute coronary syndrome (ACS) due to the imbalance in oxygen supply and consumption (3).

In the largest registry published on acute myocardial infarction (AMI) in cancer patients, the most common types of cancer associated with ACS were as follows: lung, prostate and breast cancer. Lung cancer was associated with the highest rates of in-hospital mortality and major adverse cardiovascular and cerebrovascular complications (MACCEs). In addition to this higher ischaemic risk, cancer patients also have a higher bleeding risk; colon cancer presented the highest risk of major bleeding (8).

Thus, the interaction between cancer and ACS is frequent and extremely complex, which is reflected in the higher mortality of cancer patients in this scenario (9, 10). Alterations in oxidative metabolism play important roles in the pathophysiological mechanisms underlying cancer and heart disease, especially in elderly patients. The concentrations of antioxidant enzymes are reduced in elderly subjects and may explain the reduced cardiac tolerance to oxidative stress, thus favouring the development of cardiovascular alterations. Additionally, elderly patients are more susceptible to developing cancer, and high oxidative stress during their lifespan may be an explanation for this (11).

Therapeutic decisions for these patients must involve a multidisciplinary approach, in which the clinician, oncologist, interventional cardiologist and surgeon must consider the cancer prognosis, risk of bleeding (tumour bleeding or coagulopathies), planned cancer therapy (post-chemotherapy thrombocytopaenia or oncological surgical procedures) and risk of thrombotic events. These definitions are essential for the proper prescription of anticoagulation or dual antiplatelet therapy and for deciding which treatment strategy (clinical, percutaneous, or surgical) is appropriate for each patient. This review aims to summarise the main aspects of patients with cancer and acute coronary syndrome to assist in the management, which is sometimes challenging, of these patients.

## Cancer and ACS

Cancer patients present high arterial and venous thrombotic risk (12, 13). It has been shown that at 6 months, cancer patients have more than twice the risk of arterial thromboembolism than non-cancer patients (4.7 vs. 2.2%). Most of these patients had myocardial infarction (2.0% in cancer patients vs. 0.7% in those without cancer) (14). In this same study, the types of cancers that presented the highest rates of arterial thromboembolism were lung, gastric, and pancreatic cancers (8.3, 6.5, and 5.9%, respectively). The mortality in 30 days was high in cancer patients (17.6%) when compared with that in patients without cancer (11.6%) (14).

A procoagulant state was even more prominent in the study by Brenner et al. Despite a course of anticoagulation, 8.7% patients developed a recurrence of venous thromboembolism (VTE), and 1.1% developed arterial events. Arterial thrombosis occurs earlier than the recurrence of venous thromboembolism in the follow-up after the first VTE event (36 vs. 97 days, *p* < 0.01). At 30 days of follow-up, 59% of patients with arterial events died, and the mortality associated with acute myocardial infarction (AMI) was also considerably high (40%) (15).

The procoagulant state in cancer patients is secondary to multiple mechanisms. Cancer cells present some procoagulant properties [such as procoagulant proteins (tissue factor), microparticles (MPs), coagulation factors and fibrinolysis proteins], and there is a strong interaction of platelets and endothelial cells (16). Cancer cells have two possible ways of predisposing to platelet activation: (1) direct adhesion of the cancer cell to platelets and (2) the production of molecules that lead to platelet activation by tumour cells (i.e., interleukin-6, ADP, thrombin, matrix metalloproteinases). Another potential mechanism that can explain the higher thrombosis risk is that cancer cells can activate the endothelium through adherence to endothelial cells or by the production of proinflammatory cytokines, such as thrombomodulin I, tissue factor, von Willebrand factor, selectins, and fibrinolysis proteins (PAI-1) (16).

The challenge of treating a procoagulant state (with either antiplatelet or anticoagulation therapy) is that the risk of bleeding cannot be neglected in cancer patients. For years, there was an important concern of the risk of bleeding due to thrombocytopaenia in cancer patients. Initial studies showed that cancer patients had worse prognosis than the general population, with a 1-year survival rate of only 26% (4). In this study, thrombocytopaenia (platelet count <100,000) was present in 73% of the patients. In a recently published meta-analysis, Long et al. observed that the use of dual antiplatelet therapy (DAPT) after percutaneous coronary intervention (PCI) in thrombocytopaenic patients was associated with any major bleeding [odds ratio (OR) 1.67, 95% CI: 1.42–1.97; *p* = 0.00001], including gastro-intestinal bleeding and haemorrhagic stroke (17). In patients with cancer and VTE on anticoagulant therapy, bleeding was the main cause of mortality (15).

In addition, the most frequent cause of gastrointestinal bleeding in patients receiving antithrombotic therapy is related to cancer (18). Gastrointestinal, urological and gynaecological tumours often present with active bleeding as initial symptoms. Patients with glioma have an increased risk of spontaneous intracranial haemorrhage (19). Rarely, some patients can present with ACS due to vascular compression or invasion by metastatic cardiac tumours (20). The central illustration summarises the main aspects of ACS in cancer patients.

## Cardiotoxicity: Chemotherapy and Radiotherapy

Myocardial ischaemia among cancer patients can manifest in several ways, such as AMI, until sudden cardiac death (SCD) (21). Many chemotherapeutic drugs can induce ischaemia, and the mechanism of action is variable. Usually, these mechanisms have direct action on the vessels, such as a vasospastic effect causing endothelial injury or acute arterial thrombosis (22). Long-term cancer treatment may predispose to metabolic changes such as dyslipidaemia, and previous mediastinal radiotherapy (RT) accelerates the atherosclerotic process (22). Table 1 summarises common chemotherapeutics that may induce AMI and are correlated with its main mechanisms of action.

**Table 1:** Correlations among chemotherapy, AMI incidence, and pathophysiology.

**Chemotherapeutic**	**AMI**	**Pathophysiological**
**agents**	**incidence**	**mechanisms**
Antimetabolites	0.1–10%	Vasospasm
5-FU		
Capecitabine		
Gemcitabine		
Anti-microtubule agents	0.2–4%	Vasospasm
Paclitaxel		
Docetaxel		
Vinca alkaloids	–	Vasospasm
Vincristine		
Vinblastine		
Alkylating agents	~ 2%	Acute thrombosis
Cisplatin		
Cyclophosphamide		
VEGF inhibitors		Acute thrombosis
Bevacizumab	1–3.8%	
Sorafenib	1.7%	
Sunitinib	1.4%	
Tyrosine kinase inhibitors		
Nilotinib	~ 8%	Accelerated atherosclerosis
Ponatinib	~ 2%	
Miscellaneous		
Interferon–α	Common	Endothelial dysfunction
Bortezomib	Rare	Unclear
Carfilzomib		

*5-FU, 5-fluorouracil; AMI, acute myocardial infarction; VEGF, vascular endothelial growth factor*.

Coronary vasoreactivity and consequently ACS presentations depend on the intensity and duration of vasoconstriction, and clinical manifestations vary from AMI to malignant ventricular arrhythmias (21). Among chemotherapeutic drugs, several have been listed as potential causes of coronary vasospasm, such as 5-fluorouracil (5-FU), capecitabine, paclitaxel, gemcitabine, rituximab and sorafenib (21).

Chemotherapy can precipitate type I AMI. Some drugs, such as cisplatin and vinca alkaloids, have a direct toxic effect on endothelial cells that predisposes them to the erosion and rupture of atherosclerotic plaques (21). Among cancer patients, several factors, such as infection and anaemia, induce tachycardia, which increases myocardial demand or the hypotension and hypoxaemia that reduce myocardial reserve (22). These imbalances between oxygen supply and consumption are substrates that cause type II AMI.

### Acute Vasospasm

Fluoropyrimidines (5-FU and capecitabine) and anti-microtubule agents (taxanes and vinca alkaloids) are the classes of chemotherapies most related associated with acute vasospasm (23). Considering only documented myocardial ischaemia, the incidence in patients receiving 5-FU may be as high as 10% and is influenced by dose and the time of administration (21). Taxanes can cause myocardial ischaemia, with an incidence ranging from 0.2 to 4%, and preexisting CAD might be a risk factor (24, 25).

The regulation of coronary vascular tone depends on the vasodilators released by the endothelium (26). However, the change in endothelial function does not seem to be the main cause of vasospasm mediated by 5-FU. The activation of intracellular signaling pathways that control vascular smooth muscle cell tone via protein kinase C mediated by 5-FU causes hyperreactivity and coronary spasm (21).

Myocardial ischaemia can manifest from asymptomatic ST segment changes on electrocardiography (ECG) through to angina or MI (23). Vasospasm tends to occur at sites of thrombus and plaque formation; thus, preexisting CAD remains a risk factor for 5-FU-related vasospastic angina. Other chemotherapies, such as bevacizumab, have a synergistic effect on vascular complications (23).

### Acute Thrombosis

Cisplatin agents, vascular endothelial growth factor (VEGF) signaling pathway inhibitors and cyclophosphamide predispose patients to AMI due to acute thrombosis (21). Cisplatin and cyclophosphamide are alkylating agents used in several types of solid and haematological tumours (22).

Cisplatin treatment is associated with thromboembolic events. Among arterial events, the incidence of AMI was 1.18%. These events occur mainly in the first 100 days after starting treatment (27). In the 20-year follow-up of testicular cancer survivors, the risk of developing CAD among patients treated with RT and chemotherapy was 5.3 times greater than that in the surgical group. Among survivors, the incidence of all atherosclerotic events was 8.0%. Coronary events, including AMI and unstable angina, occurred in 5.6%, with the highest incidence in the chemotherapy and RT group (28). Gietema et al. demonstrated that cisplatin can be detected in the blood even after 10 years of treatment, which explains the greater risk of developing CAD (29).

A coronary thrombosis secondary to cisplatin is mediated by platelet aggregation. Cisplatin treatment stimulates von Willebrand factor production by the endothelium stimulating platelet aggregation. Other mechanisms influenced by cisplatin, such as increased tumour necrosis factors, the formation of free radicals and decreased prostacyclin synthesis, cause intravascular platelet aggregation and predispose patients to thrombosis (30). Other potential mechanisms of ACS in patients during cisplatin treatment are related to hypomagnesaemia, which increases intracellular calcium, causing vasoconstriction and myocardial ischaemia due to the precipitation of the coronary vasospasm (30).

VEGF is a ligand present in the cell membrane that regulates signaling pathways useful for endothelial function, such as proliferation, muscle relaxation by nitric oxide and resistance to stress-induced apoptosis (31). It is estimated that the incidence and risk of ischaemic heart disease among patients treated with bevacizumab was 1 and 2.49%, respectively (32). In metastatic cancer, bevacizumab increased incidence of arterial thromboembolic events from 1.7 to 3.8% (33). Ranpura et al. showed that bevacizumab increased the risk of cardiac ischaemic events by 2.14 times (34).

A recent meta-analysis including 10,255 patients demonstrated that the risk of arterial thrombotic events in patients treated with sunitinib and sorafenib was 3.03 times greater than that in the control group (35). The inhibition of VEGF signaling pathways induces cardiotoxicity through mechanisms such as endothelial dysfunction, procoagulant status and arterial thrombosis (22). A reduction in nitric oxide production results in vasoconstriction and platelet aggregation, contributing to arterial thrombotic events (34).

### Accelerated Atherosclerosis

In chronic myeloid leukaemia, the translocation of the Bcr-Abl gene is responsible for the formation of the Philadelphia chromosome, and multitargeted TKIs act to inhibit this translocation. Nilotinib and ponatinib are second- and third- generation TKIs, respectively, and both are associated with accelerated atherosclerosis. The most common cardiovascular events during treatment include rapidly progressive peripheral arterial occlusive disease and acute ischaemic events. The accelerated atherosclerotic process generates plaques that may be responsible for coronary obliteration and cause AMI (21).

In one larger retrospective study involving 81 patients receiving nilotinib, the incidence of AMI was 7.5% (23). In the analysis of 82 patients treated with nilotinib, the cumulative incidence of atherosclerotic events at 48 months was 8.5%, which was higher among patients with elevated cardiovascular risk (36). In a study that evaluated the effectiveness of ponatinib in the treatment of CML, treatment-related adverse cardiovascular events were observed in 2% (37).

### Miscellaneous

Bortezomib is one of the key components of therapy for patients with multiple myeloma, and the association of ischaemic heart disease with bortezomib is unclear. The inhibition of proteasome activity by bortezomib should contribute to myocardial ischaemia by reducing the proliferation of endothelial progenitor cells useful in angiogenesis and decreasing the production of NO. Bortezomib also causes atherosclerotic plaque instability due to increased apoptosis (38).

Interferon-α is used to treat leukaemia, lymphomas, and melanoma. The main cardiotoxicities include fluctuations in blood pressure, arrhythmias and ischaemia. Some authors have suggested that the incidence of myocardial ischaemia induced by interferon-α is similar to that of 5-FU (39, 40).

### Radiotherapy

Radiotherapy, especially supradiaphragmatic radiotherapy, may be associated with a higher incidence of myocardial ischaemia. Pathophysiological mechanisms include endothelial injury, thrombosis and plaque rupture (22). In a 5-year assessment Hodgkin lymphoma (HL) survivors, the cumulative incidence of coronary heart disease was directly proportional to the dose of radiotherapy that the heart was exposed to (41). Another trial showed that a cumulative incidence of CVD up to 50% in long-term follow-up of HL survivors (42). A retrospective cohort study showed that patients with breast cancer and CAD undergoing radiotherapy had a 1.49-fold higher incidence of ACS (43).

### ACS in Patients With Active Hematologic Malignancies

Patients with active hematologic malignancies usually present several conditions that difficult the management of ACS, such as: thrombocytosis, thrombocytopenia, anaemia, infection, renal and hepatic dysfunction, bleeding and thrombosis. The incidence ACS in hospitalized patients with active lymphoma or leukaemia range from 1.4 to 11.2% (44, 45).

In a retrospective analysis, Park et al. observed that cardiovascular risk factors were common in patients who developed ACS with active hematologic malignancies (45). In this cohort, most patients present complex coronary artery disease with coronary angiography showed intracoronary thrombus (33%), severe coronary artery disease (50%), and mild or moderate coronary artery disease (16.7%)(45). Despite the diagnosis of ACS, only half of patients received antiplatelet, anticoagulant and/or statin therapy. The use was extremely low in those not referred to the catheterization. When we applied the criteria of platelet count of 10,000 for aspirin and 30,000 for DAPT, only 58 and 27% of eligible patients received these therapies (45, 46). A multidisciplinary approach is often necessary to reinforce the importance of following the recommendations of the guidelines and to avoid unnecessary/prolonged chemotherapy interruptions.

### Management of ACS

Cancer patients present several challenges when they are admitted with acute coronary syndrome. They are generally older and have more comorbidities and a greater extent of coronary disease. In addition, as mentioned above, haematological, and blood coagulation changes require good planning of clinical and interventional treatment when indicated (8). It is important to have a multidisciplinary approach balancing the risks of ischaemic and haemorrhagic events with cancer risk.

**Figure 1:**
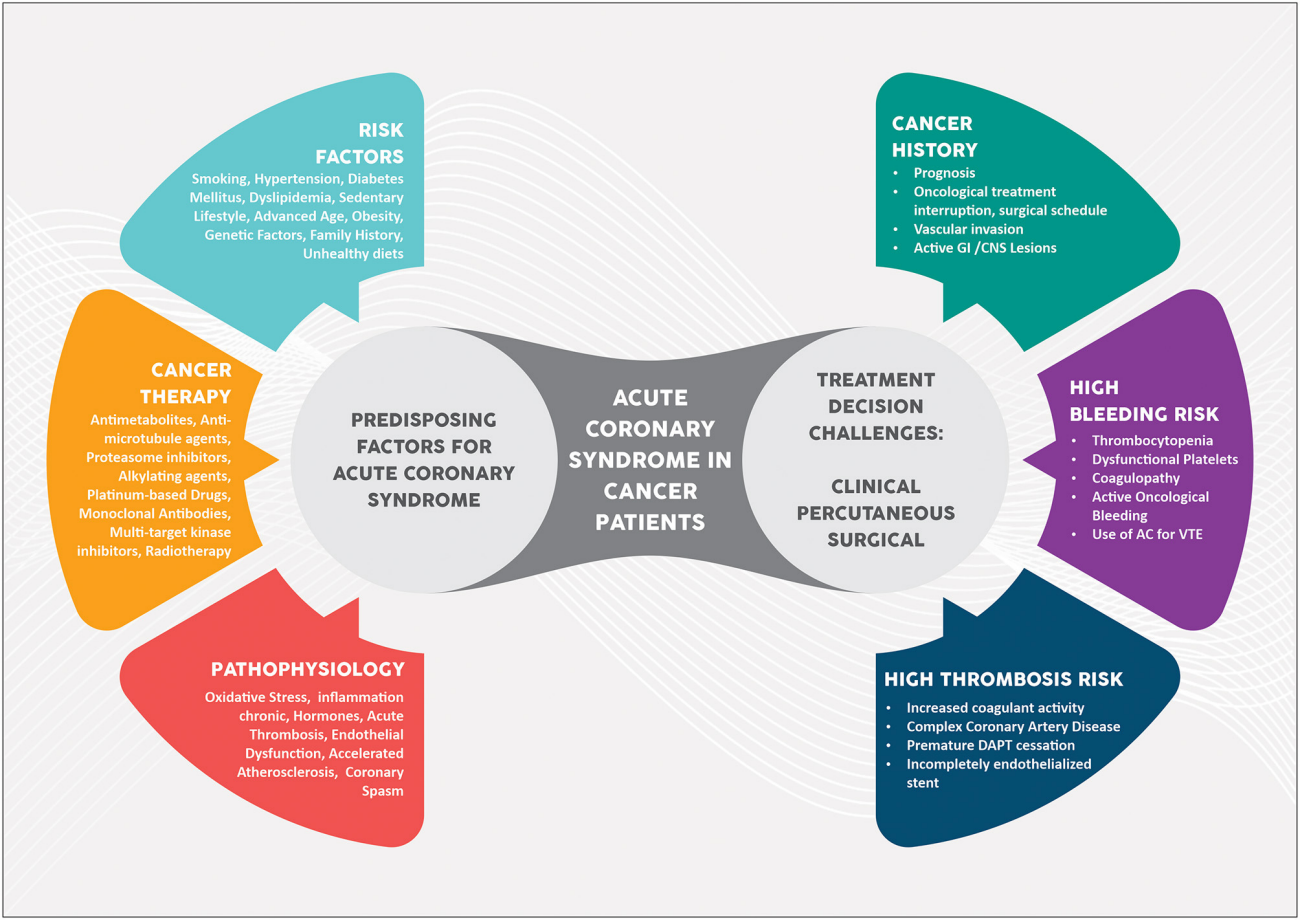
Central illustration. Acute coronary syndrome in cancer patients. AC, anticoagulation; CNS, central nervous system; DAPT, dual antiplatelet therapy, GI, gastrointestinal; VTE, venous thromboembolism.

Malignancy is considered an independent predictor of increased risk of repeated revascularization and intrastent thrombosis in patients with prior percutaneous coronary intervention (PCI)(8). Moreover, it has been shown that there is a higher atherosclerotic burden (as assessed by a higher SYNTAX score) with a high prevalence of complex lesions frequently located in the proximal segments of the coronary tree (47, 48). Figure 1 summarizes the risk factors, pathophysiology and therapeutic management of ACS in cancer patients.

### Pharmacological Treatment

There is considerable debate with regard to optimal medical therapy limitations in cancer patients suffering from ACS. Current medical guidelines favour a case-based approach (22). Balancing between bleeding and thrombotic risk is key to achieving the best clinical outcomes. A landmark study from the US National Inpatient Sample (NIS) database has made clear that cancer patients with ACS were frequently treated non-invasively despite worse outcomes than when an invasive strategy was used (8).

The coexistence of a thrombotic milieu and thrombocytopaenia offers a good practical view of the dilemma posed in this high-risk group of patients. A well-designed retrospective study on haematologic patients made the case for a reviewed contraindication for the use of aspirin. Patients with severe thrombocytopaenia (defined < 50.000) who received aspirin for ACS had improved survival compared with those who did not (92 vs. 70% at 7 days, 72 vs. 33% at 30 days, and 32 vs. 13% at 1 year; log rank *p* = 0.008). The mean platelet count was 31.000 ± 12.000. It is important to mention that these individuals did not have active bleeding (49).

Current guidelines are not unanimous in terms of the timing of DAPT and DAPT schemes for cancer patients (46, 50–53). A multidisciplinary approach (cardiologist, cardiac surgeon, interventional cardiologist, oncologist and haematologist) should be considered for the choice of an individualized treatment. The main problem in deciding the antiplatelet regimen is the lack of data to guide decisions on the type and duration of DAPT given the omission of cancer patients from most randomised trials. Furthermore, cancer is not a variable considered in DAPT prediction models (PRECISE-DAPT and DAPT scores)(50). Most recommendations consider cancer patients to be at a high risk of bleeding but do not consider their higher risk of ischaemic events. More recently, trials on antiplatelet monotherapy have shown favourable clinical outcomes in PCI patients (54–56). This may be an appealing approach for CAD in oncologic patients but requires proper validation in this population.

**Figure 2:**
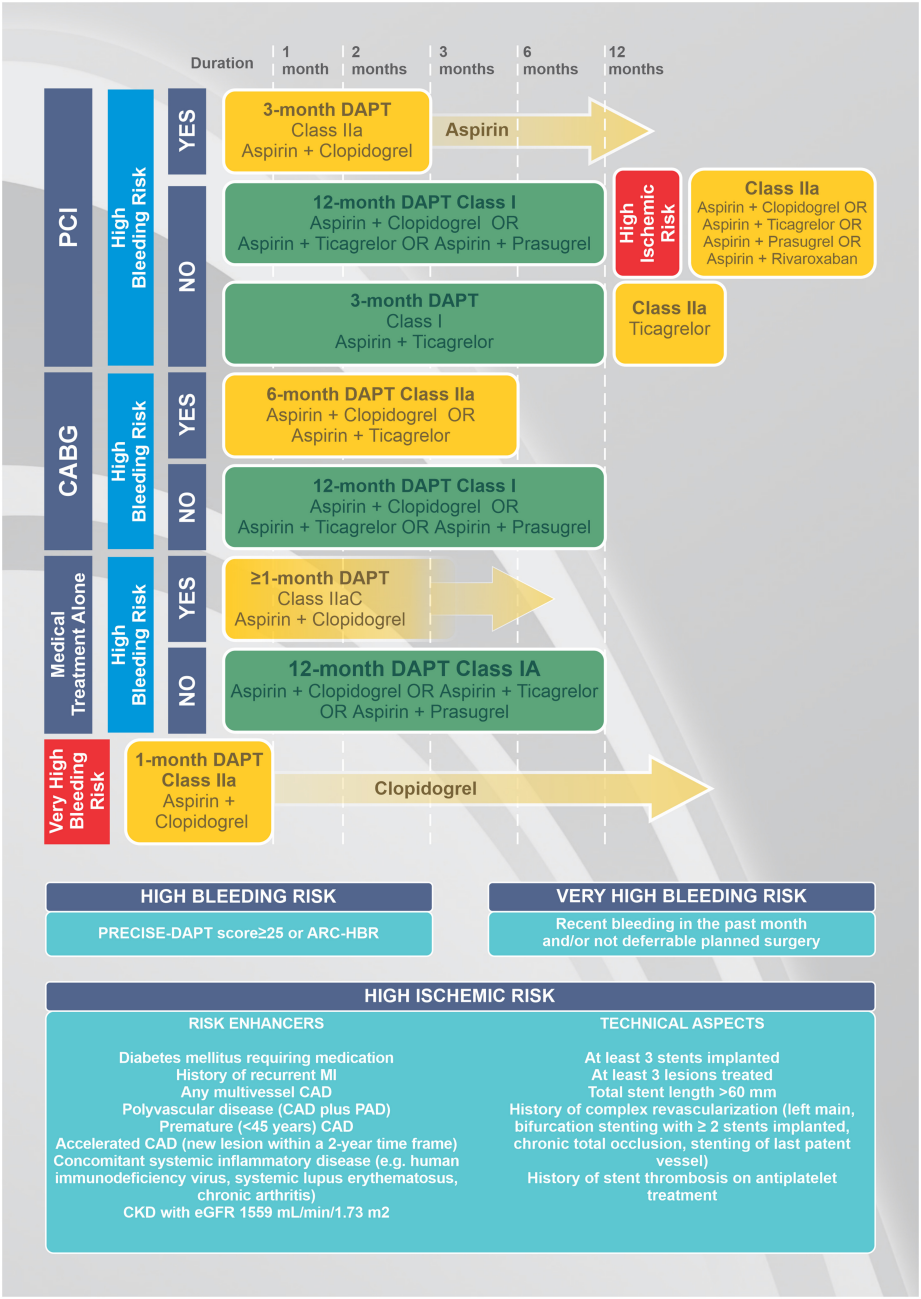
Algorithm for a dual antiplatlet therapy (DAPT) scheme and timing in patients with acute coronary syndrome according to the treatment and bleeding risk. PCI, percutaneous coronary intervention; CABG, coronary artery bypass grafting surgery; ARC-HBR, Academic Research Consortium High Bleeding Risk; PRECISE-DAPT, PREdicting bleeding Complications In patients undergoing Stent implantation and subsEquent Dual Anti Platelet Therapy; CAD, coronary artery disease; PAD, peripheral artery disease. Modified from Valgimigli et al. (50), Costa et al. (60), Collet et al. (65).

Importantly, compared with patients with stable CAD, patients with ACS are at increased thrombotic risk, warranting treatment with more potent, longer-duration antiplatelet therapy (51). The prescription of more potent platelet inhibitors, in addition to aspirin, in cancer patients suffering from myocardial infarction, was addressed in the Bern PCI Registry; in addition to them being less frequently prescribed (Cancer 30.6%vs No Cancer 41.6%; *p* < 0.001), there was not an association with increase in bleeding outcomes (57). Figure 2 provides a summary of the recommendations on DAPT schemes and duration according to the bleeding risk in ACS patients.

### Invasive Management

Currently, there are no randomised clinical studies that have evaluated the risks and benefits of clinical and interventional treatments in patients with cancer presenting with acute coronary syndromes. These patients are frequently excluded from clinical trials.

It has been shown that patients diagnosed with active cancer have lower rates of invasive management in ACS (coronary angiography, PCI and coronary artery bypass surgery) than patients without active cancer (8). The decision between CABG and PCI should take into account the coronary anatomical complexity, ability to provide complete coronary revascularization, need for an oncological surgery, and bleeding risk (including DAPT).

#### Percutaneous Coronary Intervention (PCI)

PCI is currently considered the most common type of coronary revascularization, especially in the context of ACS (8). It is the gold standard treatment for ST segment elevation acute myocardial infarction and is recommended over fibrinolysis within proper timeframes (58). For patients with refractory ischaemia, malignant arrythmias and favourable anatomy, PCI is also the therapy of choice most of the time because of the ability to reestablish coronary flow (58). For patients with more stable ACS, PCI still is a less invasive strategy and may be attractive for patients with ACS and systemic manifestations of cancer. Some anatomical factors may increase the odds of ischaemic events in patients undergoing PCI, such as three vessel disease, bifurcation with 2 stents, total stent length >60 mm, left main bifurcation PCI, saphenous graft PCI, and inability to provide complete revascularization (46, 50, 51, 53, 59–61). Another factor that must be taken into account is that the use of DAPT after PCI may postpone invasive procedures to treat cancer.

Traditionally, most of cancer patients were treated with bare-metal stents (BMS) due to concern for increased bleeding risk and expectant need for cancer-directed surgery. However, cancer patients usually present several risk factors for restenosis and stent thrombosis, such as diabetes, smoking and chronic inflammatory state. The drug-eluting stents (DES) have proven to reduce the risk of restenosis and stent thrombosis as compared with BMS. And, the newer-generation stent technology demonstrates the efficacy and safe of shorter duration of DAPT treatment. Thus, the current recommendation is that the DES is the stent of choice for cancer patients (62).

In cases of thrombocytopaenia secondary to cancer, careful vascular access and haemostasis (post-procedure) ensures greater safety and a lower incidence of adverse outcomes. There is no absolute platelet count that is a contraindication for cardiac catheterisation. Some well-established protocols recommend that this number should be at least 40,000 (so most interventional procedures are performed safely and without blood clotting abnormalities). When this count is below 50,000, a lower dose of unfractionated heparin (between 30 and 50 U/kg) may be considered, with the use of additional heparin if the ACT is below 250 s. The usual dose is 50–70 U/kg (46).

In the ACS setting, the use of the radial artery should be prioritized as the access site for PCI (Class I) (58). It has been demonstrated that, compared with transfemoral PCI, transradial PCI produces a clear reduction in adverse clinical outcomes, mainly driven by bleeding-related events. As cancer patients are considered to be at a high bleeding risk, radial access has the potential to be even more beneficial.

Intravascular imaging (intravascular ultrasound and optical coherence tomography) should be used to optimize stent implantation, assuring adequate stent expansion, apposition and a lack of edge dissection (46).

#### Coronary Artery Bypass Grafting (CABG)

Overall, CABG should be the preferred strategy for coronary revascularization for patients with complex multivessel coronary artery disease (63). In a recent study, Guha et al. evaluated CABG in cancer patients, and they observed that although the number of surgical procedures has decreased in recent years, the proportion of cancer patients who have undergone these procedures has increased (7 vs. 12.6%) (64). Surgical results are similar in cancer patients and patients without cancer, with similar mortality rates (0.9 vs. 1%), despite increased major bleeding (4.5 vs. 15.3%), and stroke (0.9 vs. 1.5%)(64).

For patients with cancer, it is important to discuss hybrid surgical procedures, staged or simultaneous coronary artery bypass grafting (CABG) and tumour resection. The best timing, strategy and possibility to use cardiopulmonary bypass are still under scientific discussion.

## Conclusions

Acute coronary syndrome in cancer patients is a frequent complication during the course of the disease and remains an important cause of mortality in these patients. Understanding the oncology history and therapy and estimating the embolic and bleeding risks are essential for proper management. The individualisation of treatment and a multidisciplinary approach are essential for a successful outcome.

## Author Contributions

LH, IC, and CC conducted the study conceptualisation, writing and review of the manuscript. FA, DC, VS, and MC participated in the writing and reviewing of the manuscript. All authors have read and approved the final version of the manuscript.

## Conflict of Interest

The authors declare that the research was conducted in the absence of any commercial or financial relationships that could be construed as a potential conflict of interest.
